# Transcriptomic Analysis of Ciguatoxin-Induced Changes in Gene Expression in Primary Cultures of Mice Cortical Neurons

**DOI:** 10.3390/toxins10050192

**Published:** 2018-05-10

**Authors:** Juan Andrés Rubiolo, Carmen Vale, Andrea Boente-Juncal, Masahiro Hirama, Shuji Yamashita, Mercedes Camiña, Mercedes R. Vieytes, Luis M. Botana

**Affiliations:** 1 Departamento de Farmacología, Farmacia e Tecnoloxía Farmacéutica, Facultade de Veterinaria, Universidade de Santiago de Compostela, 27002 Lugo, Spain; ja.rubiolo@usc.es (J.A.R.); andreaboente@gmail.com (A.B.-J.); 2 Department of Chemistry, Graduate School of Science, Tohoku University, Sendai 980-8578, Japan; hirama@m.tohoku.ac.jp (M.H.); syamashita@itsuu.or.jp (S.Y.); 3 Departamento de Fisiología, Facultad de Veterinaria, Universidad de Santiago de Compostela, 27002 Lugo, Spain; merchi.camina@usc.es (M.C.); mmercedes.rodriguez@usc.es (M.R.V.); 4 Departamento de Zoología, Genética y Antropología Física, Facultade de Veterinaria, Universidade de Santiago de Compostela, 27002 Lugo, Spain

**Keywords:** ciguatoxin, ciguatera, cortical neuron, gene, tetrodotoxin

## Abstract

Ciguatoxins are polyether marine toxins that act as sodium channel activators. These toxins cause ciguatera, one of the most widespread nonbacterial forms of food poisoning, which presents several symptoms in humans including long-term neurological alterations. Earlier work has shown that both acute and chronic exposure of primary cortical neurons to synthetic ciguatoxin CTX3C have profound impacts on neuronal function. Thus, the present work aimed to identify relevant neuronal genes and metabolic pathways that could be altered by ciguatoxin exposure. To study the effect of ciguatoxins in primary neurons in culture, we performed a transcriptomic analysis using whole mouse genome microarrays, for primary cortical neurons exposed during 6, 24, or 72 h in culture to CTX3C. Here, we have shown that the effects of the toxin on gene expression differ with the exposure time. The results presented here have identified several relevant genes and pathways related to the effect of ciguatoxins on neurons and may assist in future research or even treatment of ciguatera. Moreover, we demonstrated that the effects of the toxin on gene expression were exclusively consequential of its action as a voltage-gated sodium channel activator, since all the effects of CTX3C were avoided by preincubation of the neurons with the sodium channel blocker tetrodotoxin.

## 1. Introduction

Ciguatoxins are polyether marine toxins known to activate voltage-gated sodium channels [1,2] and cause one of the most widespread forms of nonbacterial food poisoning, named ciguatera. Ciguatera fish poisoning is a seafood-borne illness caused by the consumption of fish contaminated with ciguatera toxins, produced by marine dinoflagellates of the genus *Gambierdiscus*. Until recently, this food poisoning was endemic in several tropical and subtropical areas; however, its occurrence is increasing worldwide and several cases have recently appeared in Europe [3,4,5,6,7] and it is also slowly spreading in Australia [8,9,10,11]. Among other symptoms, in humans, ciguatera fish poisoning is characterized by neurological alterations that may last for several months or even years, including paraesthesia, headache, weakness, cold allodynia, and sensory abnormalities such as pruritus, arthralgia, myalgia, and dental pain [12,13]. In the search for the cellular mechanisms underlying the long-lasting neurological changes produced by ciguatoxins in neurons, we have described that acute exposure of cortical neurons to the synthetic ciguatoxin CTX3C caused a rapid membrane depolarization and increased the amplitude of miniature inhibitory postsynaptic currents (mIPSCs), whereas it decreased the amplitude of miniature excitatory postsynaptic currents (mEPSCs). These data indicated that acute toxin exposure could activate mechanisms that tend to suppress electrical activity by shifting the balance between excitatory and inhibitory synaptic transmission towards inhibition in order to compensate for the enhanced neuronal depolarization caused by the toxin [14]. In the same cellular model, we have also demonstrated that a larger exposure (30 min) of cortical neurons to CTX3C produced a rapid upregulation of the immediate early genes *Arc* and *Egr*, which are known to be increased by neuronal activity and are implicated in the regulation of synaptic homeostasis [15,16]. On the other hand, after 24 h exposure of cortical neurons to CTX3C, the toxin induced synaptic scaling, and its effects, besides the well-known shift in sodium channel activation to more negative voltages and membrane potential depolarization, included a decrease in neuronal firing activity and an increased frequency, but decreased amplitude of mEPSCs, which was linked to a reduced expression of *N*-methyl-d-aspartic acid (NMDA) and alpha-amino-3-hydroxy-5-methyl-4-isoxazole propionate (AMPA) receptor subunits and prevented by the voltage-gated sodium channel blocker tetrodotoxin (TTX) [17]. All these results indicate that the rapid effects induced by ciguatoxins in neurons may have long-lasting neurological effects on brain function.

Previous studies have shown that in the blood of mice, the Pacific ciguatoxin-1 (P-CTX-1, a.k.a CTX1B) at 264 ng/kg induced an acute immune response altering cytokine expression, cluster of differentiation (CD) markers, and both inflammatory and anti-inflammatory genes after 1, 4, and 24 h exposure of mice to the toxin by the intraperitoneal route [18]. At the same dose, P-CTX1 caused mainly upregulation of cytochrome P450 genes in the liver 24 h after intraperitoneal injection to mice [19]. In addition, the toxin downregulated immediate early genes such as *Fos*, *Jun*, and early growth response isoforms in the mouse brain, while it upregulated genes related with stress such as glucocorticoid responsive genes, and also decreased interleukin (IL)-1β expression in the brain 4 h after intraperitoneal injection [20]. Brevetoxins are marine polyether toxins that share with ciguatoxins a common site of action on the voltage-gated sodium channels [2] and induced only limited changes in brain gene expression after intraperitoneal injection to mice [21]. Regardless of how, a relationship between neuronal depolarization and gene expression has been established before, since the sodium channel activator veratridine increased the mRNA levels for nerve growth factor in primary hippocampal neurons in culture [22], and this effect was prevented by the sodium channel blocker TTX. On the other hand, tetrodotoxin itself has recently been shown to alter gene expression in undifferentiated mouse Neuro 2A cells in culture, but in this system, 24 h exposure of the cells to 10 nM TTX induced up- or downregulation of 43 genes that were not associated with a relevant pathway [23].

Since synthetic ciguatoxin CTX3C has been shown to have a profound effect on neuronal transmission in mice primary cortical neurons [14,17], here we aimed to identify the genes involved in the functional effects of ciguatoxins in neurons and their dependence on the sodium channel activation caused by the toxin. Thus, the current study was designed with the purpose of identifying neuronal genes and brain metabolic pathways altered by CTX exposure and whose identification could allow future research and even treatment of ciguatera. Gene ontology (GO) and Kyoto Encyclopedia of Genes and Genomes (KEGG) molecular pathway analysis were performed to identify possible relationship of the differentially expressed genes with specific biological themes.

## 2. Results

**Figure 1:**
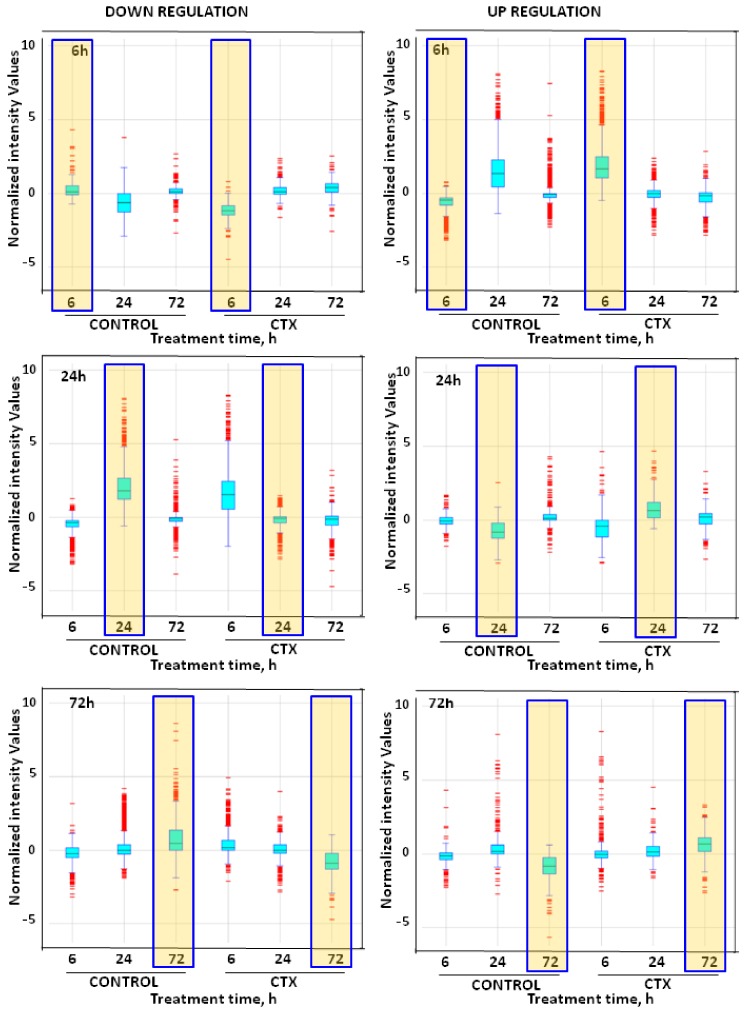
Box-and-whisker plot of increased and decreased gene transcripts in cells treated with 5 nM CTX3C (CTX) during 6, 24, or 72 h versus control cells. Yellow-shaded boxes show the assay time analyzed in each plot. Changes in transcription between treatments for each time assayed were determined by ANOVA and fold change. Genes with *p* < 0.05 (*n* = 3) and a fold change ≥ 2 were considered significant.

In this work, neuronal gene changes in response to CTX3C were investigated. Array data after 6, 24, and 72 h of exposure of primary cortical neurons to the toxin were filtered, plotted, and clustered to examine potential genes and metabolic pathways involved in the neurologic effects of the toxin. Transcriptomic analysis of cultured neurons exposed to 5 nM CTX3C for 6, 24, and 72 h showed that the toxin altered gene expression differently as time elapsed. After 6 h, CTX-treated neurons showed 2456 upregulated genes, while another 315 appeared downregulated. After 24 h, CTX-induced genes decreased to 469 and those downregulated increased to 2141. Finally, after 72 h, 412 genes appeared upregulated and another 473 downregulated (Figure 1).

**Figure 2:**
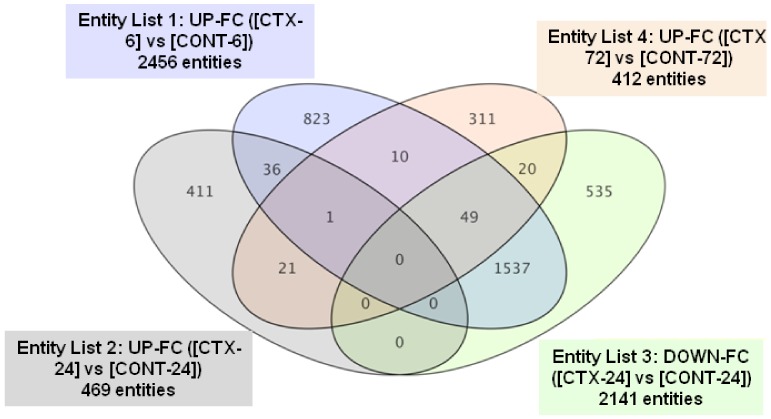
Venn diagram for CTX3C-induced genes in cortical neurons after 6, 24, or 72 h, together with those repressed after 24 h of CTX3C treatment. Color coding points to the individual conditions plotted. Intersections represent common genes affected in the corresponding conditions being compared. UP-FC: Upregulated-Fold Change; DOWN-FC: Downregulated-Fold Change.

Many genes (1537) that appeared induced by CTX after 6 h were repressed after 24 h, compared to the expression observed in control cells (see Supplementary Table S1 for the gene ID of genes shifting from up- to downregulation between 6 and 24 h), and only one gene was upregulated at the three time points assayed. Only 36 genes induced after 6 h of CTX treatment appeared upregulated at 24 h, while another 10 appeared unchanged after 24 h, but upregulated after 72 h. Of the 469 genes upregulated after 24 h, only 22 remained in the same state after 72 h, a time point that showed 412 upregulated genes. Most of the genes induced in each time point evaluated were exclusive for the time point analyzed (Figure 2).

**Figure 3:**
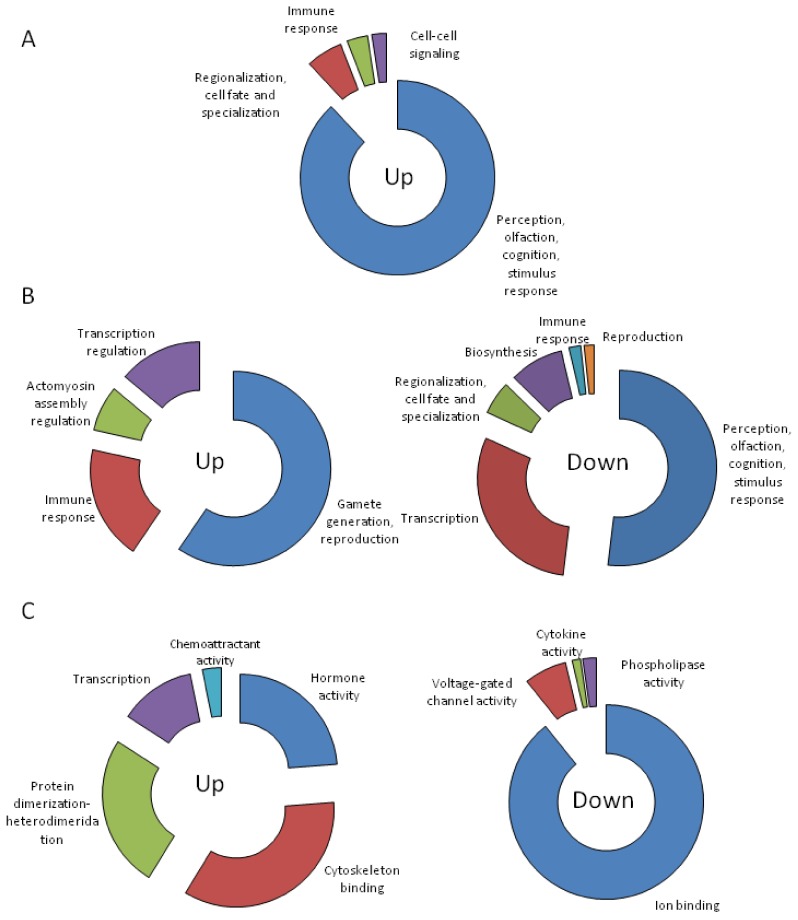
Biological processes significantly up- and downregulated in cortical neurons treated with 5 nM CTX3C during 6 h (**A**), 24 h (**B**), or 72 h (**C**). See Supplementary Table S1 for detailed information.

Gene ontology analysis of CTX-induced and repressed genes determined that after 6 h, biological processes involved in perception (olfaction, cognition, and response to stimulus), cell fate, immune response and cell–cell signaling were induced. No significant (*p* < 0.05) category was obtained for downregulated genes (Figure 3A and Supplementary Table S1). After 24 h, the immune response still appeared induced, together with biological processes related to cytoskeleton assembly, transcription regulation, and gamete generation. Many biological processes that were induced after 6 h of treatment such as perception (olfaction, cognition, and response to stimulus) and cell fate as well as some components of the immune response appeared downregulated after 24 h. Together with these, biological processes involved in transcription and biosynthesis also appeared downregulated after 24 h (Figure 3B and Supplementary Table S1). Cells treated with CTX for 72 h showed upregulated biological processes involved in hormone activity, cytoskeleton binding, protein–protein interaction, transcription, and chemoattraction. Downregulated processes involved mainly ion binding with the related voltage-gated channel activity. Interestingly, after 72 h, the genes related with phospholipase activity were also repressed (Figure 3C and Supplementary Table S1).

KEGGS pathways analysis indicated that a 6-h treatment with CTX negatively affected axon guidance while inducing olfactory transduction, among other effects (Table 1). After 24 h, olfactory transduction appeared downregulated together with several immune-related metabolic processes. Also, contrary to that observed after 6 h, axon guidance was induced after 24 h. At this last time, two other induced pathways were detected (primary immunodeficiency and purine metabolism). At the longest incubation period tested, CTX increased mitogen-activated protein kinase (MAPK) signaling pathways that lead to proliferation, differentiation, and inflammation, while decreasing other pathways involved in signaling such as that of GnRH (gonadotropin releasing hormone), the VEGF (vascular endothelial growth factor) pathway that regulates arachidonic acid (AA) metabolism, the Fc epsilon RI (high affinity receptor for immunoglobulin E) which promotes inflammation and cytokine production, the Nucleotide-binding and oligomerization domain (NOD)-like receptor that is involved in the release of proinflammatory cytokines, and the phosphatidylinositol I pathway. Furthermore, long-term depression (LTD), natural killer-mediated cytotoxicity, and lipid metabolism were downregulated after 72 h exposure of cortical neurons to CTX3C (Table 1).

**Figure 4:**
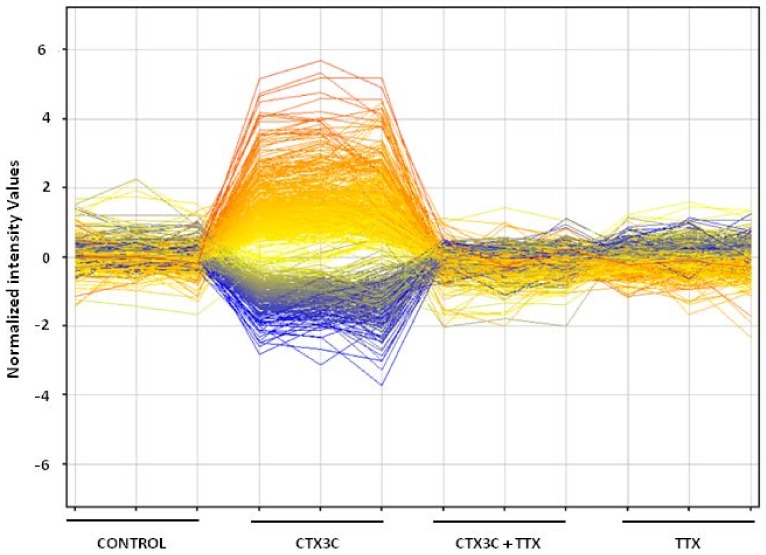
Profile plot of significantly up- and downregulated genes in cortical neurons after 24 h treatment with 5 nM CTX3C, 5 nM CTX3C in combination with TTX (5 min pretreatment), and TTX alone. Lines represent expression profiles for transcripts plotted for each treatment.

Finally, and since we have previously demonstrated that the functional alterations caused by CTX3C in primary cortical neurons were completely blocked by the sodium channel blocker tetrodotoxin [17], we aimed to determine if the CTX-induced transcriptional changes on neurons were exclusively dependent on its effect on the voltage-gated sodium channels. Therefore, primary cortical neurons were initially exposed to TTX for 5 min and then treated with CTX for another 24 h in the presence of TTX. When compared to cultures treated with CTX alone, the TTX pretreatment completely abolished the effect of CTX on neuronal gene expression. TTX by itself produced no changes on the neurons’ transcriptome (Figure 4).

## 3. Discussion

Ciguatera is a severe form of a widespread food poisoning with important neurological features. A type of the toxins involved in ciguatera are ciguatoxins, which act through activation of voltage-gated sodium channels [1,2] causing neurological long-lasting alterations [3,12,13]. Ciguatera occurrence was initially limited to tropical and subtropical areas, but nowadays, ciguatera affects a very large and diverse population in previously nonendemic areas such as the USA, Canada, and even Europe [5,7,24,25]. Although ciguatera is rarely fatal, overall, the global number of human poisonings is estimated to be 50,000–500,000 cases per year [12]. We have previously shown that the synthetic ciguatoxin CTX3C at 5 nM has profound effects on neuronal function in primary cultures of cortical neurons both after acute and chronic administration [14,17,26]. In this work, we aimed to characterize the effects of this toxin in cultured neurons evaluating the transcriptional response of primary cortical neurons to the toxin at different times in culture. Here, we show that initially CTX3C upregulated more than 2000 genes after 6 h of exposure of cortical neurons to the toxin, while many of those genes were repressed after 24 h of treatment. This finding is in agreement with our previous functional studies on the effects of CTX3C in cortical neurons, since the neuronal depolarization and the decrease in electrical activity observed after an acute exposure of neurons to the toxin was partially restored after a 24 h treatment, indicating that compensatory mechanisms were involved in the neuronal effects of the toxin, probably in an attempt to restore neuronal activity to normal levels [14,17].

Among the genes downregulated after 6 h of treatment, the only downregulated pathway was axon guidance, which was not associated with any relevant biological process. However, after 24 h, seven pathways were downregulated. At this time point, one downregulated biological process was related with cell surface receptor-linked signal transduction, in which 111 genes were identified, mostly associated with olfactory (OlfR) and vomeronasal (Vmn) receptor mRNA. Downregulated genes were also associated with the V1 olfactory receptor protein and with the mu1 opioid receptor mRNA transcript. Activation of the mu1 opiod receptor is required for perpetuation of hypothermia [27], and since intraperitoneal administration of P-CTX-1 induces a pronounced hypothermia that lasts for about 7 h [20], it is suggested that the downregulation of the mu1 receptor could try to compensate this effect.

Among the downregulated pathways after 72 h treatment, the first downregulated pathway was the gonadotropin-releasing hormone (GnRH), whose receptor is coupled to G proteins leading to activation of the intracellular protein kinase C (PKC) pathway. Signaling downstream of protein kinase C leads to transactivation of the epidermal growth factor (EGF) receptor and activation of mitogen-activated protein kinases (MAPKs), including extracellular-signal-regulated kinase (ERK), Jun N-terminal kinase (JNK), and p38 MAPK. Active MAPKs translocate to the nucleus, resulting in activation of transcription factors and rapid induction of early genes. Thus, the downregulation of the GnRH signaling pathway described here will ultimately result in the downregulation of immediate early genes, which is in agreement with the previously described effects of PCTX-1 in the mouse brain that caused the downregulation of immediate early genes such as *Fos*, *Jun*, and early growth response (*Egr*) isoforms [20]. However, a rapid upregulation of the immediate early genes *Arc* and *Egr1*, in a manner dependent on the activating effects of CTX3C on voltage-dependent sodium channels, was also previously described [17]. However, in this work, the pathway was downregulated only after 72 h of treatment, while the previous in vivo study described downregulation of immediate early genes already after 1 h of treatment [20], a fact probably attributable to the lower toxicity of CTX3C versus P-CTX-1, which led the European Food Security Authority to establish a toxicity equivalency factor of 1 for P-CTX1 and 0.2 for P-CTX3C [28]. This observation was also found in our laboratory, since oral administration of CTX3C to mice at doses of 1000 ng/kg did not elicit any toxicity signs. Other downregulated genes within this pathway were those for calmodulin, phospholipase A2 (PLA2) and phospholipase C, MAP kinase 6, and gonadotropin-releasing hormone receptors, as well as protein tyrosine kinase 2. Among the downregulated genes involved in different signaling pathways, the phospholipase A2 genes (group IIC, IID, and IIE) were most prevalent. These phospholipases are involved in inflammatory responses through the increase in arachidonic acid release and prostaglandin synthesis, therefore its downregulation is expected to have an overall anti-inflammatory effect. According to this, several genes involved in inflammatory responses have also found to be downregulated in the brain after acute exposure of mice to P-CTX-1 [20], although this in vivo effect was attributed mainly to the hypothermia elicited by the toxin. Downregulation of phospholipase A2 genes is also involved in neuroprotection against synaptic damage [29] and could also be related with the decrease in excitatory synaptic activity elicited by the toxin in cortical neurons [14,17], since phospholipase A2 is related with *N*-methyl-d-aspartic acid (NMDA) receptor activation [30]. Again, after 72 h of treatment, five genes involved in long-term depression were downregulated; among those, three genes codified phospholipase A2, one gene phospholipase C, and the fifth gene was the guanine nucleotide-binding protein alpha 13. Some evidence indicates that changes in synaptic function during LTD are the result of modifications of postsynaptic currents mediated by the alpha-amino-3-hydroxy-5-methyl-4-isoxazole propionate (AMPA) subtype of glutamate receptors, and we have previously demonstrated that 24 h exposure of cortical neurons to CTX3C decreased AMPA receptor subunit expression and the amplitude of miniature postsynaptic excitatory currents [17]. However, the results presented here indicate that the signaling cascades associated with the phospholipase A2 and phospholipase C enzymes could be involved in the in vitro neuronal effects of ciguatoxin. Since both the phospholipase C and phospholipase A2 pathways link long-term depression with metabotropic glutamate receptors [31,32], further studies should be designated to investigate the chronic effects of ciguatoxin treatment on neuronal metabotropic glutamate receptors and phospholipase activity and expression. Moreover, the dysfunction of genes related with phospholipase A2 can lead to stroke and neuronal alterations [33]. In cultured primary cortical neurons, the stimulation of ionotropic glutamate receptors by NMDA has been shown to activate cPLA2 and arachidonic acid release and to mediate the neurotoxic effects of NMDA [30]. Therefore, as pointed out above, the decrease in the NMDA receptor expression caused by the toxin [17] could be related to the downregulation of the phospholipase A2 genes.

At 6 h post-treatment, several upregulated genes were found, but many of them were downregulated after 24 h. At 24 h, five of the upregulated genes were involved in axon guidance, specifically the semaphorin 6 and 4 genes, that participate in nervous system development and the Ephrin *(Eph)* receptors B and A5 genes. The Eph family of molecules comprises the largest group of receptor tyrosine kinases. These molecules have been shown to play important roles in many physiological functions including tissue segmentation, angiogenesis, axon guidance, and neural plasticity [34]. After 72 h, upregulated genes include the brain derived neurotrophic factor gene, which encodes the protein BDNF, a member of the nerve growth factor family that participates in the growth, differentiation, and survival of specific types of developing neurons in the central nervous system and in regulating synaptic plasticity in the brain [35]. This fact is likely related to sodium channel activation, since a previous report also showed that the sodium channel activator veratridine increased the mRNA levels for nerve growth factor in primary hippocampal neurons in culture and this effect was prevented by application of TTX [22].

To determine if the changes in gene expression patterns observed here were only derived from the binding of ciguatoxin to voltage-gated sodium channels, coincubations with TTX were performed. This last toxin acts by blocking voltage-gated sodium channels, and TTX by itself had no effects on gene expression in this neuronal model. However, we observed that a brief 5-min incubation with TTX was enough to completely abolish the transcriptional changes produced by ciguatoxin. These results indicate that the whole effects of this molecule on gene expression are consequence of its agonist effect on voltage-gated sodium channels.

In summary, we have identified several relevant genes and pathways related to the effect of CTX3C on neurons, which were mediated exclusively by its effect of activating voltage-gated sodium channels, which can help to clarify the short- and long-term effects of these molecules in the course of an intoxication.

## 4. Materials and Methods

### 4.1. Toxins and Drugs Used

The standard of CTX3C was synthesized by Dr. Masahiro Hirama following previously described procedures and dissolved at a stock concentration of 10 μM in DMSO. Following dilutions were performed in deionized water, and the toxin was used at a final concentration of 5 nM (51 ng/mL) in the culture medium. The solvent concentration (DMSO) in the culture medium was 0.005% (*v/v*). The voltage-dependent sodium channel blocker tetrodotoxin (TTX) was purchased from CIFGA (Lugo, Spain) and used at a final concentration of 0.5 µM in the culture medium.

### 4.2. Primary Cultures of Cortical Neurons

Primary cortical neurons were obtained from embryonic day 16−18 Swiss mice following previously described procedures [14,17,26,36]. All protocols were approved by the University of Santiago de Compostela Institutional Animal Care and Use Committee. Briefly, pregnant mice were euthanized using a CO_2_ mice chamber and the cerebral cortices of the fetuses were removed and dissociated by mild trypsinization, followed by mechanical trituration in a DNase solution (0.004% *w*/*v*) containing a soybean trypsin inhibitor (0.05% *w*/*v*). The cells were suspended in Neurobasal medium supplemented with 1% B-27 supplement (Invitrogen), 5 mM l-glutamine, and 1% penicillin/streptomycin. The cell suspension was seeded in 6 or 12 multiwell plates precoated with poly-d-lysine, and the cell culture was kept in a 95% air and 5% CO_2_ atmosphere at 37 °C. Culture medium was replaced every 3−4 days. Neurons were employed between 7 and 10 days in culture. All data were obtained in parallel from drug-treated and age-matched sister control cultures. After 7 days in culture, cortical neurons were treated with 5 nM CTX 3C or vehicle (DMSO/water) either alone or in the presence of 0.5 µM TTX. Changes in gene expression were evaluated at 6, 24, and 72 h after treatment. For each array, three biological replicates (each containing three to four technical replicates) from three independent cultures were used.

### 4.3. RNA Extraction, Purification, Quantification, and Integrity Determination

The Aurum^TM^ Total RNA Mini Kit (Bio-Rad, Hercules, CA, USA) was used to obtain RNA for microarray assays, following the manufacturer instructions. RNA concentration was determined with a NanoDrop 2000 (Thermo Scientific, Waltham, MA, USA) and integrity was confirmed with a Bioanalyzer 2100 (Agilent, Santa Clara, CA, USA) using the kit RNA 6000 nanoreagents (Agilent).

### 4.4. Microarray Assay and Analysis

To obtain fluorescently labeled RNA for microarray hybridization, we used the Low RNA Input Linear Amp Kit (Agilent Technologies, Santa Clara, CA, USA) and the Quick-Amp Labeling Kit (Agilent Technologies) and followed the manufacturer instructions. Briefly, Cyanine-3 (Cy3) labeled cRNA was prepared from 1 µg RNA using the Quick-Amp Kit (Agilent Technologies), followed by RNeasy column purification (Qiagen, Hilden, Germany). Dye incorporation and cRNA yield were checked with a Nanodrop (Thermo Scientific) spectrophotometer. Cy3-labelled cRNA (1.5 µg; specific activity >9.0 pmol Cy3/µg cRNA) was fragmented at 60 °C for 30 min in a reaction volume of 25 µL containing 10× Blocking Agent (Agilent Technologies), nuclease-free water, and 25× Fragmentation Buffer (Agilent Technologies). On completion of the fragmentation reaction, 25 µL of 2× GE Hybridization Buffer (HI-RPM0) (Agilent Technologies) was added to the fragmentation mixture which was hybridized to 8X60K SurePrint G3 Agilent Whole Mouse Genome Oligo Microarrays (G4852A) containing 39,430 features, for 17 h at 65 °C in a rotating Agilent hybridization oven. After hybridization, microarrays were washed for 1 min at room temperature with GE Wash Buffer 1 and 1 min at 37 °C with GE Wash Buffer 2 (Agilent Technologies). After washing, the microarrays were scanned with a MS200 scanner (Roche, Basel, Switzerland) using one color scan setting (scan resolution 2 µm). Each slide, containing 8 arrays, was scanned using a single image mode without autogain and with 100% gain for acquisition. The scanned images were extracted with Feature Extraction Software 11.5.1.1 (Agilent Technologies) using default parameters (protocol GE1-QCMT_11.5 and Grid: 028005_D_F20131202).

After extraction, differential gene expression as a consequence of time in culture and response to medium change was determined using GeneSpring software (Version 11.5, Agilent Technologies, Santa Clara, CA, USA, 2010).

### 4.5. Statistical Analysis

Gene lists were analyzed for differentially expressed (DE) genes using one-way ANOVA set at *p* < 0.05, with a fold change cutoff = 2. Data mining for significant altered pathways and ontological categories at the biological process level 5 were performed with the DAVID Bioinformatics Database [37,38].

## Figures and Tables

**Table 1:** KEGGS pathways significantly altered by CTX in mouse primary neuron cultures.

Term	Count	%	*p* Value	Genes
**Downregulated Pathways**				
**6 h**				
mmu04360: Axon guidance	4	3.77	0.02060781	NM_001126047, NM_010143, NM_007937, NM_199241
**24 h**				
mmu04740: Olfactory transduction	45	6.52	7.04 × 10^−6^	NM_146825, NM_146826, NM_146827, NM_146348, NM_146366, NM_146829, NM_146347, NM_147075, NM_146843, NM_147034, NM_146807, NM_147055, NM_146698, NM_146729, NM_146871, NM_147002, L08075, NM_146796, NM_146277, NM_001011531, NM_146776, NM_146523, NM_146975, NM_146895, NM_146526, NM_146525, NM_146524, NM_146691, NM_146873, NM_001011866, NM_146529, NM_147006, NM_207254, NM_146508, NM_146747, NM_146903, NM_146926, NM_146689, NM_146944, Y15524, NM_182714, NM_146644, NM_207132, NM_146311, NM_028910
mmu04650: Natural killer cell-mediated cytotoxicity	8	1.16	0.0270638	NM_016659, NM_010649, NM_008333, NM_018729, NM_013793, NM_199022, NM_013794, AF296427, NM_014194, AF296435, NM_053152, NM_001160402
mmu05332: Graft-versus-host disease	5	0.72	0.0500518	NM_016659, NM_008204, NM_013793, NM_014194, NM_009855, NM_053152, NM_201611
mmu04640: Hematopoietic cell lineage	6	0.87	0.05094991	NM_001082960, NM_181858, AF296427, NM_007641, AF296435, NM_001033228, NM_001043317
mmu04060: Cytokine–cytokine receptor interaction	11	1.59	0.06837512	NM_010228, NM_008501, NM_030712, NM_009915, NM_009916, NM_008333, NM_009835, NM_001161842, NM_007719, NM_008176, NM_021380
mmu05416: Viral myocarditis	6	0.87	0.07518154	NM_009989, NM_008204, AK149002, AF296427, NM_009855, AF296435, NM_201611
mmu05320: Autoimmune thyroid disease	5	0.72	0.09434947	NM_008333, NM_008204, AF296427, NM_009855, AF296435, NM_201611
**72 h**				
mmu04912: GnRH signaling pathway	7	1.52	0.0076469	NM_027416, NM_008868, AK086722, NM_012044, NM_011109, NM_010323, AK036896
mmu04370: VEGF signaling pathway	6	1.30	0.01140765	NM_008868, NM_177320, NM_012044, NM_011109, NM_016791, NM_001130409
mmu04664: Fc epsilon RI signaling pathway	6	1.30	0.01548986	NM_008868, NM_008355, NM_177320, AK086722, NM_012044, NM_011109
mmu00590: Arachidonic acid metabolism	6	1.30	0.01625387	NM_008868, NM_019455, NM_012044, NM_206537, NM_011109, AK049084
mmu04270: Vascular smooth muscle contraction	7	1.52	0.02034802	NM_027416, NM_008868, NM_010303, NM_175441, NM_012044, NM_011109, AK036896
mmu04650: Natural killer cell mediated cytotoxicity	7	1.52	0.02188488	NM_177320, NM_016659, NM_008333, NM_010493, NM_199022, NM_014194, NM_053152, NM_016791
mmu04730: Long-term depression	5	1.09	0.03976529	NM_008868, NM_010303, NM_012044, NM_011109, AK036896
mmu00592: Alpha-linolenic acid metabolism	3	0.65	0.04050261	NM_008868, NM_012044, NM_011109
mmu04070: Phosphatidylinositol signaling system	5	1.09	0.04512758	NM_027416, NM_177320, AK038544, AK036896, AK039149
mmu00591: Linoleic acid metabolism	4	0.87	0.04889228	NM_008868, NM_012044, NM_206537, NM_011109
mmu00512: O-Glycan biosynthesis	3	0.65	0.08379939	NM_145218, NM_009177, NM_011371
mmu04621: NOD-like receptor signaling pathway	4	0.87	0.09955218	NM_145857, NM_007464, NM_001033367, NM_008176
**Upregulated Pathways**				
**6 h**				
mmu04740: Olfactory transduction	41	7.69	5.49 × 10^−6^	NM_146825, NM_146827, NM_146348, NM_146829, NM_146700, NM_146347, NM_146849, NM_146345, NM_147075, NM_207224, NM_147034, AK136433, NM_147055, NM_146698, NM_146681, NM_146861, NM_147049, NM_146890, NM_146323, L08075, NM_146796, NM_001011531, NM_146776, NM_146523, NM_146714, NM_146895, NM_147061, NM_146691, NM_146873, NM_146670, NM_147006, NM_207254, NM_146508, NM_146591, NM_010974, NM_146903, NM_146689, Y15524, NM_206823, NM_146647, NM_146749, NM_028910
mmu05332: Graft-versus-host disease	5	0.94	0.03245265	NM_177635, NM_016659, NM_008204, NM_013793, NM_014194, NM_009855
mmu05320: Autoimmune thyroid disease	5	0.94	0.06317509	NM_177635, NM_008333, NM_008204, AF296427, NM_009855
mmu04640: Hematopoietic cell lineage	5	0.94	0.09834101	NM_001082960, AF296427, NM_007641, NM_001033228, NM_001043317
**24 h**				
mmu04360: Axon guidance	5	2.07	0.05239993	NM_001126047, NM_027470, NM_010143, NM_007937, NM_199241
mmu05340: Primary immunodeficiency	3	1.24	0.05722627	NM_001040691, NM_013482, NM_017395
mmu00230: Purine metabolism	5	2.07	0.08886492	NM_030187, NM_011864, NM_173029, AF038896, NM_028093
**72 h**				
mmu04010: MAPK signaling pathway	10	4.03	0.00220468	NM_001111030, NM_007540, NM_009370, NM_176933, NM_007922, NM_001081023, NM_013642, NM_008002, NM_011246, NM_010234
mmu05200: Pathways in cancer	8	3.23	0.06220855	NM_001111030, NM_013598, NM_009370, NM_001122733, NM_008485, NM_008002, NM_011836, NM_010234

## Supplementary Materials

The following are available online at http://www.mdpi.com/2072-6651/10/5/192/s1, Table S1: Biological processes altered by CTX identified by gene ontology analysis.
