# Supplementary material for: Transcriptomic Analysis of Ciguatoxin-Induced Changes in Gene Expression in Primary Cultures of Mice Cortical Neurons
Source: Toxins (Basel). 2018 May 10;10(5):192. doi: 10.3390/toxins10050192 (PMC5983248; doi:10.3390/toxins10050192)
Supplement: Supplementary file 1 [file toxins-10-00192-s001.zip › toxins-294031-supplementary.pdf]

Supplementary Materials: Transcriptomic Analysis of Ciguatoxin-Induced Changes in Gene Expression in Primary Cultures of Mice Cortical Neurons

Juan Andrés Rubiolo, Carmen Vale, Andrea Boente-Juncal, Masahiro Hirama, Shuji Yamashita, Mercedes Camiña, Mercedes R. Vieytes and Luis M. Botana

Supplementary table 1. Biological processes altered by CTX identified by gene ontology analysis.

Category	Term	Count	P Value	Genes
**Down-regulated biological processes**				
**24 hs**				
GOTERM_BP_FAT	GO:0007186~G-protein coupled receptor protein signaling pathway	93	5,76E-10	NM_146348, NM_134180, NM_146347, NM_146843, NM_146807, NM_147055, NM_001011831, NM_146698, NM_029525, NM_146871, NM_146609, NM_030739, NM_007719, NM_146796, NM_001011531, NM_001104573, NM_153067, NM_001104575, NM_146523, NM_001166710, NM_198961, NM_146895, NM_146526, NM_009835, NM_009314, NM_146525, NM_146524, NM_001166719, NM_001165944, NM_001011866, NM_146529, NM_147006, NM_207254, NM_146903, NM_146944, NM_182714, NM_001105065, NM_001105067, NM_001104562, NM_146825, NM_146826, NM_205810, NM_146366, NM_001011859, NM_146827, NM_030712, NM_203490, NM_207546, NM_009915, NM_146829, NM_181817, NM_009916, NM_001011807, NM_146982, NM_207543, XM_001475614, NM_147075, NM_147034, NM_001166727, NM_053093, NM_203492, NM_146729, NM_001011519, NM_176996, NM_147002, NM_146277, NM_001011811, NM_146776, NM_146975, AK143547, NM_175669, NM_134231, NM_001105152, NM_053220, NM_001105156, NM_146691, NM_134173, NM_146873, NM_134436, NM_134177,

				NM_001011740, NM_146508, NM_134175, NM_134169, NM_146747, XM_001481325, NM_134167, NM_001104540, NM_206868, NM_001039652, NM_146689, NM_146926, NM_133720, Y15524, NM_007538, NM_146644, NM_207132, NM_146311, NM_028910
GOTERM_BP_FAT	GO:0007166~cell surface receptor linked signal transduction	111	3,41E-09	NM_134180, NM_146843, NM_029525, NM_146871, NM_145467, NM_001082960, NM_026505, NM_007719, NM_146796, NM_001104573, NM_008006, NM_001104575, NM_146523, NM_001166710, NM_146526, NM_009314, NM_009835, NM_146525, NM_146524, NM_001166719, NM_001011866, NM_010204, NM_146529, NM_147006, NM_146903, NM_182714, NM_001105065, NM_001105067, NM_001104562, NM_146825, NM_146826, NM_146827, NM_030712, NM_203490, NM_009915, NM_146829, NM_009916, NM_001011807, NM_146982, NM_147075, NM_001166727, NM_053116, NM_053093, NM_203492, NM_146729, NM_007950, NM_147002, NM_146277, NM_022657, NM_001011811, NM_146776, NM_010228, NM_146975, AK143547, NM_053220, NM_146691, NM_134173, NM_146873, NM_134436, NM_134177, NM_001011740, NM_144955, NM_146508, NM_134175, NM_134169, XM_001481325, NM_134167, NM_206868, NM_146926, NM_146689, NM_007538, NM_207132, NM_146311, NM_001033228, NM_028910, NM_146348, NM_146347, NM_146807, NM_147055, NM_001011831, NM_146698, NM_146609, NM_145745, NM_030739, NM_001011531, NM_153067, AK138305, NM_198961,

				NM_146895, NM_001165944, NM_207254, AK012979, NM_030614, NM_146944, NM_205810, NM_146366, NM_001011859, NM_207546, NM_181817, NM_207543, XM_001475614, NM_147034, NM_001011519, NM_176996, NM_001043317, NM_175669, NM_134231, NM_001105152, NM_001105156, NM_031180, NM_146747, NM_001104540, NM_001039652, NM_133720, NM_008011, Y15524, NM_146644
GOTERM_BP_FAT	GO:0007600~sensory perception	66	2,60E-06	NM_146348, NM_146347, AK016832, NM_146843, NM_146807, NM_001142747, NM_147055, NM_001011831, NM_146698, NM_013624, NM_146871, NM_146609, NM_146086, NM_146796, NM_001011531, NM_146523, NM_080450, NM_146895, NM_146526, NM_146525, NM_146524, NM_001011866, NM_001165944, NM_146529, NM_147006, NM_207254, NM_146903, NM_146944, NM_182714, AK044695, NM_146825, NM_146826, NM_146366, NM_146827, NM_001011859, NM_009915, NM_146829, NM_001011807, NM_146982, XM_001475614, NM_147075, NM_147034, NM_146729, NM_001011519, L08075, NM_146277, NM_001011811, NM_146776, NM_146975, AK143547, NM_053220, NM_146691, NM_001011740, NM_134177, NM_146508, NM_146747, XM_001481325, AK044345, NM_146689, NM_146926, Y15524, NM_007538, NM_146644, NM_207132, NM_146311, NM_028910
GOTERM_BP_FAT	GO:0050890~cognition	66	1,55E-05	NM_146348, NM_146347, AK016832, NM_146843, NM_146807, NM_001142747, NM_147055, NM_001011831, NM_146698, NM_013624, NM_146871, NM_146609, NM_146086, NM_146796, NM_001011531, NM_146523, NM_080450, NM_146895, NM_146526, NM_146525, NM_146524, NM_001011866,

				NM_001165944, NM_146529, NM_147006, NM_207254, NM_146903, NM_146944, NM_182714, AK044695, NM_146825, NM_146826, NM_146366, NM_146827, NM_001011859, NM_009915, NM_146829, NM_001011807, NM_146982, XM_001475614, NM_147075, NM_147034, NM_146729, NM_001011519, L08075, NM_146277, NM_001011811, NM_146776, NM_146975, AK143547, NM_053220, NM_146691, NM_001011740, NM_134177, NM_146508, NM_146747, XM_001481325, AK044345, NM_146689, NM_146926, Y15524, NM_007538, NM_146644, NM_207132, NM_146311, NM_028910
GOTERM_BP_FAT	GO:0007608~sensory perception of smell	53	2,75E-05	NM_146348, NM_146347, NM_146843, NM_146807, NM_001011831, NM_147055, NM_146698, NM_146609, NM_146871, NM_146796, NM_001011531, NM_146523, NM_146895, NM_146526, NM_146525, NM_146524, NM_001011866, NM_001165944, NM_146529, NM_147006, NM_207254, NM_146903, NM_146944, NM_182714, NM_146825, NM_146826, NM_146827, NM_001011859, NM_146366, NM_146829, NM_001011807, NM_146982, NM_147075, XM_001475614, NM_147034, NM_146729, NM_001011519, NM_146277, NM_001011811, NM_146776, NM_146975, NM_146691, NM_001011740, NM_146508, NM_146747, XM_001481325, NM_146689, NM_146926, Y15524, NM_146644, NM_207132, NM_146311, NM_028910
GOTERM_BP_FAT	GO:0007606~sensory perception of chemical stimulus	55	3,97E-05	NM_146348, NM_146347, NM_146843, NM_146807, NM_001011831, NM_147055, NM_146698, NM_146609, NM_146871, NM_146796, NM_001011531, NM_146523, NM_146895, NM_146526, NM_146525, NM_146524,

				NM_001011866, NM_001165944, NM_146529, NM_147006, NM_207254, NM_146903, NM_146944, NM_182714, NM_146825, NM_146826, NM_146827, NM_001011859, NM_146366, NM_146829, NM_001011807, NM_146982, NM_147075, XM_001475614, NM_147034, NM_146729, NM_001011519, NM_146277, NM_001011811, NM_146776, NM_146975, NM_053220, NM_146691, NM_001011740, NM_134177, NM_146508, NM_146747, XM_001481325, NM_146689, NM_146926, Y15524, NM_146644, NM_207132, NM_146311, NM_028910
GOTERM_BP_FAT	GO:0050877~neurological system process	70	7,15E-05	NM_146348, NM_146347, AK149002, AK016832, NM_146843, NM_146807, NM_001142747, NM_147055, NM_001011831, NM_146698, NM_013624, NM_146871, NM_146609, NM_146086, NM_146796, NM_001011531, NM_009891, NM_146523, NM_080450, NM_146895, NM_146526, NM_146525, NM_146524, NM_001011866, NM_001165944, NM_146529, NM_147006, NM_207254, AK036325, NM_146903, NM_146944, NM_182714, AK044695, NM_146825, NM_146826, NM_146366, NM_001011859, NM_146827, NM_009915, NM_146829, NM_001011807, NM_146982, XM_001475614, NM_147075, NM_147034, NM_146729, NM_001011519, L08075, NM_146277, NM_001011811, NM_146776, NM_146975, AK143547, NM_053220, NM_146691, NM_001011740, NM_134177, NM_146508, NM_146747, XM_001481325, AK044345, NM_011428, NM_146689, NM_146926, Y15524, NM_007538, NM_146644, NM_207132, NM_146311, NM_028910
GOTERM_BP_FAT	GO:0008543~fibroblast growth factor receptor signaling pathway	6	9,72E-04	NM_030614, NM_008011, NM_031180, NM_010204, NM_022657, NM_008006
GOTERM_BP_FAT	GO:0019236~response to	10	0,001559243	NM_001166710, NM_134169, NM_134167, NM_134180,

	pheromone			NM_134231, NM_053220, NM_134173, NM_001166719, NM_134436, NM_134177, NM_134175
GOTERM_BP_FAT	GO:0008284~positive regulation of cell proliferation	16	0,007837211	NM_008501, AK037664, NM_031180, NM_010204, NM_007903, NM_011562, NM_007950, NM_176996, NM_030614, NM_145136, NM_008011, NM_008989, NM_198052, NM_009855, AK138305, NM_008006
GOTERM_BP_FAT	GO:0006355~regulation of transcription, DNA-dependent	54	0,008413424	NM_177278, AK142139, NM_008103, NM_009541, NM_145707, NM_194063, NM_001013765, BC034883, AK138305, NM_008006, NM_008501, NM_008500, NM_025684, NM_001040089, NM_010464, NM_008270, NM_199059, AK142268, NM_008273, NM_008236, NM_001110316, NM_001033638, NM_145136, NM_009803, NM_009880, NM_145710, NM_008989, NM_001005425, NM_030226, XM_001480563, NM_010455, AK021218, NM_030676, NM_001039698, NM_176996, NM_011041, NM_198052, NM_007500, NM_008593, NM_022657, AK162583, NM_010441, NM_001003666, NM_130869, NM_009719, NM_001039198, NM_019732, NM_144955, NM_181319, NM_008657, NM_011562, NM_001166584, NM_001024850, NM_139226, NM_001039688
GOTERM_BP_FAT	GO:0051252~regulation of RNA metabolic process	54	0,011146055	NM_177278, AK142139, NM_008103, NM_009541, NM_145707, NM_194063, NM_001013765, BC034883, AK138305, NM_008006, NM_008501, NM_008500, NM_025684, NM_001040089, NM_010464, NM_008270, NM_199059, AK142268, NM_008273, NM_008236, NM_001110316, NM_001033638, NM_145136, NM_009803, NM_009880, NM_145710, NM_008989, NM_001005425, NM_030226, XM_001480563, NM_010455, AK021218, NM_030676, NM_001039698, NM_176996, NM_011041,

				NM_198052, NM_007500, NM_008593, NM_022657, AK162583, NM_010441, NM_001003666, NM_130869, NM_009719, NM_001039198, NM_019732, NM_144955, NM_181319, NM_008657, NM_011562, NM_001166584, NM_001024850, NM_139226, NM_001039688
GOTERM_BP_FAT	GO:0045893~positive regulation of transcription, DNA-dependent	20	0,012737307	NM_008501, NM_130869, NM_010455, NM_008270, NM_009719, NM_030676, AK142268, AK142139, NM_009541, NM_011562, NM_001166584, NM_176996, NM_145136, NM_009880, NM_011041, NM_198052, NM_007500, NM_022657, AK138305, NM_008006
GOTERM_BP_FAT	GO:0051254~positive regulation of RNA metabolic process	20	0,013830448	NM_008501, NM_130869, NM_010455, NM_008270, NM_009719, NM_030676, AK142268, AK142139, NM_009541, NM_011562, NM_001166584, NM_176996, NM_145136, NM_009880, NM_011041, NM_198052, NM_007500, NM_022657, AK138305, NM_008006
GOTERM_BP_FAT	GO:0001708~cell fate specification	6	0,018515382	NM_176996, NM_008011, NM_007500, NM_008273, NM_144955, NM_008006
GOTERM_BP_FAT	GO:0045449~regulation of transcription	74	0,019713154	NM_011061, NM_177278, AK046648, NM_028572, AK149002, NM_029416, AK142139, NM_008103, NM_009541, NM_145707, NM_194063, NM_001013765, BC034883, NM_028679, AK138305, NM_008006, NM_008501, NM_008500, NM_025684, NM_001040089, NM_010464, NM_008270, AK142268, NM_199059, NM_008273, NM_172495, NM_008236, NM_001110316, NM_001033638, NM_016812, NM_145136, NM_145710, NM_009880, NM_009803, NM_008989, NM_009804, NM_001033281, NM_001005425, NM_027658, NM_030226, AK037664, XM_001480563, NM_010455, AK021218, NM_030676,

				NM_183140, NM_007950, NM_176996, NM_001039698, NM_011041, NM_198052, NM_007500, NM_008593, NM_022657, NM_007893, NM_010441, AK162583, AK048565, NM_130869, NM_001003666, NM_001039198, NM_009719, NM_019732, AK033609, NM_144955, NM_181319, NM_008657, AK017287, NM_172446, NM_011562, NM_001166584, NM_001024850, NM_139226, NM_001039688, NM_011050
GOTERM_BP_FAT	GO:0010557~positive regulation of macromolecule biosynthetic process	23	0,021438055	NM_008501, AK037664, NM_130869, NM_010455, NM_008270, NM_009719, NM_030676, AK142268, AK142139, NM_008657, NM_009541, NM_011562, NM_001166584, NM_007950, NM_176996, NM_145136, NM_009880, NM_011041, NM_198052, NM_007500, NM_022657, AK138305, NM_008006
GOTERM_BP_FAT	GO:0045941~positive regulation of transcription	21	0,024160439	NM_008501, NM_130869, NM_010455, NM_008270, NM_009719, NM_030676, AK142268, AK142139, NM_008657, NM_009541, NM_011562, NM_001166584, NM_176996, NM_145136, NM_009880, NM_011041, NM_198052, NM_007500, NM_022657, AK138305, NM_008006
GOTERM_BP_FAT	GO:0003002~regionalization	12	0,024972855	NM_011562, NM_011652, NM_176996, NM_010455, NM_010464, NM_009880, NM_008270, NM_198052, NM_008273, NM_144955, AK138305, NM_008657
GOTERM_BP_FAT	GO:0045935~positive regulation of nucleobase, nucleoside, nucleotide and nucleic acid metabolic process	22	0,026339383	NM_008501, NM_130869, NM_010455, NM_008270, NM_009719, NM_030676, AK142268, AK142139, NM_008657, NM_009541, NM_011562, NM_001166584, NM_007950, NM_176996, NM_145136, NM_009880, NM_011041, NM_198052, NM_007500, NM_022657, AK138305, NM_008006
GOTERM_BP_FAT	GO:0051148~negative regulation of muscle cell differentiation	3	0,026523869	NM_007950, NM_198052, NM_001033281

GOTERM_BP_FAT	GO:0007276~gamete generation	16	0,027395876	NM_177866, NM_173023, NM_010418, NM_130869, NM_009220, NM_001160135, AK129256, NM_001145038, NM_001017409, NM_001160141, NM_013694, NM_001160142, NM_029416, NM_009693, NM_001025241, NM_007950, XM_001477675, NM_001085543, NM_019445, XM_001472176, XM_001474092, NM_013932, NM_183099
GOTERM_BP_FAT	GO:0010628~positive regulation of gene expression	21	0,030836506	NM_008501, NM_130869, NM_010455, NM_008270, NM_009719, NM_030676, AK142268, AK142139, NM_008657, NM_009541, NM_011562, NM_001166584, NM_176996, NM_145136, NM_009880, NM_011041, NM_198052, NM_007500, NM_022657, AK138305, NM_008006
GOTERM_BP_FAT	GO:0031328~positive regulation of cellular biosynthetic process	23	0,031906241	NM_008501, AK037664, NM_130869, NM_010455, NM_008270, NM_009719, NM_030676, AK142268, AK142139, NM_008657, NM_009541, NM_011562, NM_001166584, NM_007950, NM_176996, NM_145136, NM_009880, NM_011041, NM_198052, NM_007500, NM_022657, AK138305, NM_008006
GOTERM_BP_FAT	GO:0051147~regulation of muscle cell differentiation	4	0,032299999	NM_007950, NM_145136, NM_198052, NM_001033281
GOTERM_BP_FAT	GO:0009891~positive regulation of biosynthetic process	23	0,034870049	NM_008501, AK037664, NM_130869, NM_010455, NM_008270, NM_009719, NM_030676, AK142268, AK142139, NM_008657, NM_009541, NM_011562, NM_001166584, NM_007950, NM_176996, NM_145136, NM_009880, NM_011041, NM_198052, NM_007500, NM_022657, AK138305, NM_008006
GOTERM_BP_FAT	GO:0051173~positive regulation of nitrogen compound metabolic process	22	0,03539263	NM_008501, NM_130869, NM_010455, NM_008270, NM_009719, NM_030676, AK142268, AK142139, NM_008657, NM_009541, NM_011562, NM_001166584, NM_007950, NM_176996, NM_145136, NM_009880, NM_011041, NM_198052, NM_007500,

				NM_022657, AK138305, NM_008006
GOTERM_BP_FAT	GO:0006955~immune response	20	0,040128114	NM_001033978, NM_008501, NM_009915, AK075670, D38613, NM_001161842, NM_194336, NM_001169153, AF296427, NM_001143689, AF296435, NM_053093, NM_153761, NM_198297, NM_008204, NM_007719, NM_008176, AF487898, NM_008428, NM_145437, NM_201611
GOTERM_BP_FAT	GO:0019953~sexual reproduction	17	0,045873743	AK143547, NM_177866, NM_173023, NM_010418, NM_130869, NM_009220, NM_001160135, AK129256, NM_001145038, NM_001017409, NM_001160141, NM_013694, NM_001160142, NM_029416, NM_009693, NM_001025241, NM_007950, XM_001477675, NM_001085543, NM_019445, XM_001472176, XM_001474092, NM_013932, NM_183099
GOTERM_BP_FAT	GO:0009952~anterior/posterior pattern formation	9	0,046987789	NM_011562, NM_011652, NM_010455, NM_010464, NM_009880, NM_008270, NM_198052, NM_008273, NM_008657
GOTERM_BP_FAT	GO:0045944~positive regulation of transcription from RNA polymerase II promoter	16	0,048341705	NM_008501, NM_010455, NM_130869, NM_008270, NM_009719, NM_030676, AK142139, NM_011562, NM_001166584, NM_176996, NM_145136, NM_009880, NM_011041, NM_007500, AK138305, NM_008006
GOTERM_BP_FAT	GO:0007601~visual perception	7	0,048354155	AK143547, NM_146086, AK044345, NM_007538, L08075, NM_001142747, AK044695
		**72 hs**		
GOTERM_BP_FAT	GO:0002821~positive regulation of adaptive immune response	4	0,016907796	NM_145857, NM_017371, NM_013632, NM_008324
GOTERM_BP_FAT	GO:0002824~positive regulation of adaptive immune response based on somatic recombination of immune receptors built	4	0,016907796	NM_145857, NM_017371, NM_013632, NM_008324

	from immunoglobulin superfamily domains			
GOTERM_BP_FAT	GO:0006665~sphingolipid metabolic process	5	0,017418438	AK016156, NM_008635, AK082974, NM_018830, NM_011371
GOTERM_BP_FAT	GO:0006643~membrane lipid metabolic process	5	0,019357366	AK016156, NM_008635, AK082974, NM_018830, NM_011371
GOTERM_BP_FAT	GO:0007269~neurotransmitter secretion	4	0,019709598	AK036325, AK149250, BC005523, NM_153579
GOTERM_BP_FAT	GO:0016042~lipid catabolic process	7	0,020969081	NM_009693, NM_008868, NM_023114, NM_053115, NM_012044, NM_011109, AK039149
GOTERM_BP_FAT	GO:0016053~organic acid biosynthetic process	7	0,026139018	NM_001081664, NM_178767, NM_019455, AK049084, AK149431, NM_008324
GOTERM_BP_FAT	GO:0046394~carboxylic acid biosynthetic process	7	0,026139018	NM_001081664, NM_178767, NM_019455, AK049084, AK149431, NM_008324
GOTERM_BP_FAT	GO:0032989~cellular component morphogenesis	12	0,026368172	NM_011652, AK082711, NM_010451, NM_008635, NM_001142732, AK149002, AF153046, NM_007936, NM_007601, NM_007868, NM_016791, NM_001113481, NM_001130409
GOTERM_BP_FAT	GO:0045926~negative regulation of growth	5	0,027166769	NM_145857, NM_007706, AK033206, AK138305, NM_001113481, NM_001130409
GOTERM_BP_FAT	GO:0044272~sulfur compound biosynthetic process	4	0,027766557	AK049084, AK149431, AK166962
GOTERM_BP_FAT	GO:0001816~cytokine production	4	0,031409388	NM_145857, NM_001033367, NM_029926, NM_008324
GOTERM_BP_FAT	GO:0043062~extracellular structure organization	7	0,033011627	NM_022315, AK149250, NM_011682, AK041037, BC005523, NM_007583, NM_001130409
GOTERM_BP_FAT	GO:0055085~transmembrane transport	14	0,034646665	NM_177866, NM_177809, NM_001039104, NM_011075, AK044260, NM_153579, NM_026200, NM_152923, NM_001006676, NM_011887, NM_198304, NM_153512, NM_001033336, NM_145551
GOTERM_BP_FAT	GO:0006687~glycosphingolipid metabolic process	3	0,036625572	AK016156, NM_008635, NM_011371
GOTERM_BP_FAT	GO:0046700~heterocycle catabolic process	4	0,037319002	NM_001164289, NM_013632, AF038896, NM_008324

GOTERM_BP_FAT	GO:0007389~pattern specification process	10	0,039825349	NM_011562, NM_011652, NM_010451, NM_010928, NM_011265, NM_010303, NM_175542, AF153046, AK084505, AK138305
GOTERM_BP_FAT	GO:0050808~synapse organization	4	0,043755313	AK149250, NM_011682, BC005523, NM_007583
GOTERM_BP_FAT	GO:0048858~cell projection morphogenesis	8	0,044330253	AK082711, NM_010451, NM_001142732, AK149002, AF153046, NM_007936, NM_007868, NM_001113481, NM_001130409
GOTERM_BP_FAT	GO:0003001~generation of a signal involved in cell-cell signaling	5	0,046043342	AK036325, AK149250, NM_016977, BC005523, NM_153579
GOTERM_BP_FAT	GO:0032735~positive regulation of interleukin-12 production	2	0,047366075	NM_145857, NM_008324
GOTERM_BP_FAT	GO:0006664~glycolipid metabolic process	3	0,047956379	AK016156, NM_008635, NM_011371
**Up-regulated biological processes**				
**6 hs**				
GOTERM_BP_FAT	GO:0007186~G-protein coupled receptor protein signaling pathway	77	1,24E-08	NM_021340, NM_146348, NM_134180, NM_146700, NM_146347, NM_146849, NM_146345, NM_147055, NM_001011831, NM_001011736, NM_020488, NM_146698, NM_146861, NM_029525, NM_147049, NM_001010830, NM_001011535, NM_007719, NM_146796, NM_001011531, NM_001104573, NM_153067, NM_146523, NM_146895, NM_147061, NM_009314, NM_001166719, NM_001165944, NM_146670, NM_147006, NM_010323, NM_207254, NM_010974, NM_146903, NM_001105065, NM_001105067, NM_001104562, NM_146825, NM_146827, NM_203490, NM_207546, NM_009915, NM_146829, XM_001475614, NM_147075, NM_207224, NM_147034, NM_020510, NM_001166727, NM_203492, NM_146681, NM_176996, NM_146890, NM_146323, NM_134213, NM_001011811, NM_146776, AK143547, NM_146714, NM_134231, NM_001105152,

				NM_001105156, NM_134173, NM_146691, NM_146873, NM_001011740, NM_134177, NM_146508, NM_134175, NM_001104543, NM_146591, NM_001105180, NM_134169, XM_001481325, NM_001104540, NM_206868, NM_001039652, NM_146689, Y15524, NM_206823, NM_146647, NM_146749, NM_028910
GOTERM_BP_FAT	GO:0007166~cell surface receptor linked signal transduction	89	4,02E-07	NM_021340, NM_146348, NM_134180, NM_146700, NM_146347, NM_146849, NM_146345, AK046903, NM_147055, NM_001011831, NM_001011736, NM_020488, NM_146698, NM_146861, NM_029525, NM_147049, NM_001010830, NM_145745, NM_145467, NM_001082960, NM_001011535, NM_007719, NM_146796, NM_001011531, NM_001104573, AK138305, NM_153067, NM_146523, NM_146895, NM_147061, NM_009314, NM_001166719, NM_001165944, NM_146670, NM_147006, NM_010323, NM_207254, NM_010974, NM_146903, NM_030614, NM_001105065, NM_001105067, NM_001104562, NM_146825, NM_146827, NM_203490, NM_207546, NM_009915, NM_146829, XM_001475614, NM_147075, NM_207224, NM_147034, NM_020510, NM_001166727, NM_203492, NM_146681, NM_176996, NM_146890, NM_146323, NM_022657, NM_134213, NM_001011811, NM_001043317, NM_146776, AK143547, NM_146714, NM_134231, NM_001105152, NM_027665, NM_001105156, NM_134173, NM_146691, NM_146873, NM_001011740, NM_134177, NM_146508, NM_144955, NM_134175, NM_001104543, NM_146591, NM_001105180, NM_134169, XM_001481325, NM_001104540, NM_206868, NM_001039652, NM_146689, NM_008011, Y15524, NM_206823,

				NM_001033228, NM_146647, NM_146749, NM_028910
GOTERM_BP_FAT	GO:0007600~sensory perception	56	5,64E-06	NM_021340, NM_146348, NM_146700, NM_146347, NM_146849, NM_146345, AK016832, NM_001011831, NM_001011736, NM_147055, NM_146698, NM_146861, NM_147049, NM_146086, NM_146796, NM_001011531, NM_146523, NM_080450, NM_146895, NM_147061, NM_001165944, NM_146670, NM_147006, NM_207254, NM_010974, NM_172621, NM_146903, AK044695, NM_146825, NM_146827, NM_146829, NM_009915, NM_147075, XM_001475614, NM_207224, NM_147034, NM_146681, NM_146890, NM_146323, L08075, NM_001038845, NM_001011811, NM_146776, AK143547, NM_146691, NM_001011740, NM_134177, NM_146508, NM_146591, NM_053077, XM_001481325, NM_146689, Y15524, NM_206823, NM_146647, NM_146749, NM_028910
GOTERM_BP_FAT	GO:0050890~cognition	56	2,70E-05	NM_021340, NM_146348, NM_146700, NM_146347, NM_146849, NM_146345, AK016832, NM_001011831, NM_001011736, NM_147055, NM_146698, NM_146861, NM_147049, NM_146086, NM_146796, NM_001011531, NM_146523, NM_080450, NM_146895, NM_147061, NM_001165944, NM_146670, NM_147006, NM_207254, NM_010974, NM_172621, NM_146903, AK044695, NM_146825, NM_146827, NM_146829, NM_009915, NM_147075, XM_001475614, NM_207224, NM_147034, NM_146681,

				NM_146890, NM_146323, L08075, NM_001038845, NM_001011811, NM_146776, AK143547, NM_146691, NM_001011740, NM_134177, NM_146508, NM_146591, NM_053077, XM_001481325, NM_146689, Y15524, NM_206823, NM_146647, NM_146749, NM_028910
GOTERM_BP_FAT	GO:0050877~neurological system process	60	6,87E-05	NM_021340, NM_146348, NM_146700, NM_146347, NM_146849, AK149002, NM_146345, AK016832, NM_001011831, NM_001011736, NM_147055, NM_146698, NM_146861, NM_147049, NM_146086, NM_146796, NM_001011531, NM_009891, NM_146523, NM_080450, NM_146895, NM_147061, NM_001165944, NM_146670, NM_147006, NM_207254, NM_010974, NM_172621, AK036325, NM_146903, AK044695, NM_146825, NM_146827, NM_146829, NM_009915, NM_147075, XM_001475614, NM_207224, NM_147034, NM_146681, NM_146890, NM_146323, L08075, NM_001038845, NM_001011811, NM_146776, AK143547, NM_146691, NM_001011740, NM_134177, NM_146508, NM_146591, NM_053077, XM_001481325, NM_146689, NM_011428, Y15524, NM_206823, NM_146647, NM_146749, NM_028910
GOTERM_BP_FAT	GO:0007608~sensory perception of smell	44	1,07E-04	NM_146825, NM_146827, NM_146348, NM_146829, NM_146700, NM_146347, NM_146849, NM_146345, NM_147075, XM_001475614, NM_207224, NM_147034, NM_001011831, NM_001011736, NM_147055, NM_146698, NM_146681, NM_146861, NM_147049, NM_146890, NM_146323, NM_146796, NM_001011531, NM_001011811, NM_146776, NM_146523, NM_146895, NM_147061, NM_146691, NM_001165944, NM_146670, NM_147006, NM_001011740, NM_207254, NM_146508, NM_146591, NM_010974, XM_001481325, NM_146903, NM_146689, Y15524, NM_206823, NM_146647, NM_146749, NM_028910

GOTERM_BP_FAT	GO:0007606~sensory perception of chemical stimulus	45	2,27E-04	NM_146825, NM_146827, NM_146348, NM_146829, NM_146700, NM_146347, NM_146849, NM_146345, NM_147075, XM_001475614, NM_207224, NM_147034, NM_001011831, NM_001011736, NM_147055, NM_146698, NM_146681, NM_146861, NM_147049, NM_146890, NM_146323, NM_146796, NM_001011531, NM_001011811, NM_146776, NM_146523, NM_146895, NM_147061, NM_146691, NM_001165944, NM_146670, NM_134177, NM_147006, NM_001011740, NM_207254, NM_146508, NM_146591, NM_010974, XM_001481325, NM_146903, NM_146689, Y15524, NM_206823, NM_146647, NM_146749, NM_028910
GOTERM_BP_FAT	GO:0019236~response to pheromone	8	0,006849656	NM_134169, NM_134180, NM_134231, NM_134173, NM_001166719, NM_134177, NM_134213, NM_134175
GOTERM_BP_FAT	GO:0003002~regionalization	11	0,016688383	NM_011562, NM_010452, NM_176996, NM_010455, NM_010464, NM_009880, NM_008270, NM_175730, NM_008273, NM_144955, AK138305
GOTERM_BP_FAT	GO:0001708~cell fate specification	5	0,037008316	NM_010861, NM_176996, NM_008011, NM_008273, NM_144955
GOTERM_BP_FAT	GO:0030500~regulation of bone mineralization	3	0,044735864	NM_001038845, NM_022657, NM_031368
GOTERM_BP_FAT	GO:0070167~regulation of biomineral formation	3	0,044735864	NM_001038845, NM_022657, NM_031368
GOTERM_BP_FAT	GO:0006955~immune response	17	0,045568646	NM_001033978, NM_021319, NM_001170333, NM_001081032, NM_009915, AK075670, NM_016850, D38613, NM_001169153, NM_194336, AF296427, NM_177635, NM_198297, NM_008204, NM_007719, AF487898, NM_009946, NM_145437
GOTERM_BP_FAT	GO:0009952~anterior/posterior pattern formation	8	0,045839525	NM_011562, NM_010452, NM_010455, NM_010464, NM_009880, NM_008270, NM_175730, NM_008273

GOTERM_BP_FAT	GO:0007267~cell-cell signaling	12	0,04712737	AK036325, AK143547, NM_080450, NM_176996, AK149002, NM_011428, NM_008011, NM_001038845, NM_009891, NM_020510, NM_008273, AK138305
		**24 hs**		
GOTERM_BP_FAT	GO:0030239~myofibril assembly	3	0,01161087	NM_009608, NM_001164171, NM_011619
GOTERM_BP_FAT	GO:0031032~actomyosin structure organization	3	0,019692913	NM_009608, NM_001164171, NM_011619
GOTERM_BP_FAT	GO:0006955~immune response	10	0,021452078	NM_009888, NM_011095, NM_133893, NM_001040691, NM_175397, NM_133200, NM_016850, NM_133211, NM_013478, NM_023476
GOTERM_BP_FAT	GO:0007283~spermatogenesis	7	0,02376532	NM_030080, NM_207680, NM_170671, NM_175643, NM_009292, NM_173029, BC090648
GOTERM_BP_FAT	GO:0048232~male gamete generation	7	0,02376532	NM_030080, NM_207680, NM_170671, NM_175643, NM_009292, NM_173029, BC090648
GOTERM_BP_FAT	GO:0002252~immune effector process	5	0,024070227	NM_009888, NM_011095, NM_001040691, NM_016850, NM_133211
GOTERM_BP_FAT	GO:0007276~gamete generation	8	0,025304878	NM_133893, NM_030080, NM_207680, NM_170671, NM_175643, NM_009292, NM_173029, BC090648
GOTERM_BP_FAT	GO:0032504~multicellular organism reproduction	9	0,026096597	NM_133893, NM_030080, NM_207680, NM_170671, NM_011680, NM_175643, NM_009292, NM_173029, BC090648
GOTERM_BP_FAT	GO:0048609~reproductive process in a multicellular organism	9	0,026096597	NM_133893, NM_030080, NM_207680, NM_170671, NM_011680, NM_175643, NM_009292, NM_173029, BC090648
GOTERM_BP_FAT	GO:0007066~female meiosis sister chromatid cohesion	2	0,034290397	NM_009292, BC090648
GOTERM_BP_FAT	GO:0010927~cellular component assembly involved in morphogenesis	3	0,038884361	NM_009608, NM_001164171, NM_011619
GOTERM_BP_FAT	GO:0006357~regulation of transcription from RNA polymerase II promoter	11	0,04169182	NM_030080, NM_010800, NM_029469, BC026991, NM_001012518, NM_017395, NM_001168502, NM_011680, AK081813, NM_011912,

				NM_139227
GOTERM_BP_FAT	GO:0051177~meiotic sister chromatid cohesion	2	0,042679218	NM_009292, BC090648
		**72 hs**		
GOTERM_BP_FAT	GO:0030324~lung development	7	0,00232002	NM_008259, NM_212452, NM_008970, NM_010728, NM_205769, NM_008002, NM_001159487
GOTERM_BP_FAT	GO:0030323~respiratory tube development	7	0,002538044	NM_008259, NM_212452, NM_008970, NM_010728, NM_205769, NM_008002, NM_001159487
GOTERM_BP_FAT	GO:0048732~gland development	9	0,002799628	NM_010828, NM_008259, NM_212452, NM_008970, NM_009370, NM_010456, NM_205769, NM_008002, NM_008700
GOTERM_BP_FAT	GO:0060541~respiratory system development	7	0,004023507	NM_008259, NM_212452, NM_008970, NM_010728, NM_205769, NM_008002, NM_001159487
GOTERM_BP_FAT	GO:0035295~tube development	10	0,004964891	NM_007540, NM_008259, NM_212452, NM_008970, NM_010728, NM_015743, NM_205769, NM_008002, NM_001159487, NM_008700
GOTERM_BP_FAT	GO:0048610~reproductive cellular process	8	0,005188178	NM_008259, NM_025727, NM_013598, NM_021879, NM_009370, NM_001122733, NM_001159487, NM_198418
GOTERM_BP_FAT	GO:0008354~germ cell migration	3	0,007501416	NM_013598, NM_009370, NM_001122733
GOTERM_BP_FAT	GO:0048070~regulation of pigmentation during development	3	0,007501416	NM_007903, NM_013598, NM_001122733
GOTERM_BP_FAT	GO:0007281~germ cell development	6	0,007720224	NM_025727, NM_013598, NM_021879, NM_001122733, NM_001159487, NM_198418
GOTERM_BP_FAT	GO:0043062~extracellular structure organization	7	0,009665215	NM_212452, NM_010728, NM_009370, NM_016907, NM_001081023, NM_008940, NM_007424
GOTERM_BP_FAT	GO:0043069~negative regulation of programmed cell death	9	0,009906034	NM_007540, NM_010828, NM_013598, NM_009129, NM_009370, NM_001122733, NM_007429, NM_007702, NM_008700
GOTERM_BP_FAT	GO:0033555~multicellular organismal response to stress	4	0,010095664	NM_007540, NM_009311, NM_001002927, NM_008102

GOTERM_BP_FAT	GO:0060548~negative regulation of cell death	9	0,010138906	NM_007540, NM_010828, NM_013598, NM_009129, NM_009370, NM_001122733, NM_007429, NM_007702, NM_008700
GOTERM_BP_FAT	GO:0032504~multicellular organism reproduction	12	0,011291423	NM_010828, NM_025727, NM_013598, NM_001039385, NM_008970, NM_021879, NM_009370, NM_001122733, NM_009061, NM_001159487, NM_013498, NM_198418
GOTERM_BP_FAT	GO:0048609~reproductive process in a multicellular organism	12	0,011291423	NM_010828, NM_025727, NM_013598, NM_001039385, NM_008970, NM_021879, NM_009370, NM_001122733, NM_009061, NM_001159487, NM_013498, NM_198418
GOTERM_BP_FAT	GO:0042127~regulation of cell proliferation	14	0,014103056	NM_007540, NM_013598, NM_009129, NM_008970, NR_001592, NM_001122733, NM_001159487, NM_008700, NM_007903, NM_008037, NM_008002, NM_021451, NM_008021, NM_011340
GOTERM_BP_FAT	GO:0007218~neuropeptide signaling pathway	5	0,014918406	NM_009311, NM_001002927, NM_013732, NM_010932, NM_009625
GOTERM_BP_FAT	GO:0003006~reproductive developmental process	9	0,015346789	NM_010828, NM_008259, NM_025727, NM_013598, NM_001039385, NM_021879, NM_001122733, NM_001159487, NM_198418
GOTERM_BP_FAT	GO:0009968~negative regulation of signal transduction	7	0,018066661	NM_153171, NM_011915, NM_020265, NM_009061, NM_009062, NM_028679, NM_007702
GOTERM_BP_FAT	GO:0043067~regulation of programmed cell death	14	0,019013797	NM_007540, NM_001111030, NM_013598, NM_009129, NM_001122733, NM_009370, NM_007429, NM_008700, NM_010828, NM_008102, NM_021451, NM_007702, NM_028679, NM_138313
GOTERM_BP_FAT	GO:0043405~regulation of MAP kinase activity	5	0,019090065	NM_013598, NM_027106, NM_001122733, NM_175093, NM_028679
GOTERM_BP_FAT	GO:0010941~regulation of cell death	14	0,019772735	NM_007540, NM_001111030, NM_013598, NM_009129, NM_001122733, NM_009370, NM_007429, NM_008700, NM_010828, NM_008102, NM_021451, NM_007702, NM_028679,

				NM_138313
GOTERM_BP_FAT	GO:0010817~regulation of hormone levels	6	0,019906047	NM_008259, NM_001039385, NM_013628, NM_205769, NM_001159487, NM_198408
GOTERM_BP_FAT	GO:0035150~regulation of tube size	4	0,020403199	NM_007903, NM_007429, NM_008102, NM_010933
GOTERM_BP_FAT	GO:0050880~regulation of blood vessel size	4	0,020403199	NM_007903, NM_007429, NM_008102, NM_010933
GOTERM_BP_FAT	GO:0008284~positive regulation of cell proliferation	9	0,022688649	NM_007903, NM_013598, NM_009129, NM_008970, NM_001122733, NM_008037, NM_008021, NM_008002, NM_008700
GOTERM_BP_FAT	GO:0003018~vascular process in circulatory system	4	0,022720082	NM_007903, NM_007429, NM_008102, NM_010933
GOTERM_BP_FAT	GO:0044087~regulation of cellular component biogenesis	5	0,023067282	NM_207267, NM_009370, NM_009635, AK020250, NM_008700
GOTERM_BP_FAT	GO:0042445~hormone metabolic process	5	0,023918651	NM_008259, NM_013628, NM_205769, NM_001159487, NM_198408
GOTERM_BP_FAT	GO:0060658~nipple morphogenesis	2	0,024139564	NM_212452, NM_008970
GOTERM_BP_FAT	GO:0060618~nipple development	2	0,024139564	NM_212452, NM_008970
GOTERM_BP_FAT	GO:0010648~negative regulation of cell communication	7	0,025995955	NM_153171, NM_011915, NM_020265, NM_009061, NM_009062, NM_028679, NM_007702
GOTERM_BP_FAT	GO:0030199~collagen fibril organization	3	0,026459378	NM_010728, NM_009370, NM_007424
GOTERM_BP_FAT	GO:0043066~negative regulation of apoptosis	8	0,026740307	NM_007540, NM_010828, NM_013598, NM_009129, NM_009370, NM_007429, NM_007702, NM_008700
GOTERM_BP_FAT	GO:0048568~embryonic organ development	8	0,027928578	NM_010828, NM_015743, NM_009370, NM_009323, NM_026571, NM_016907, NM_008002, NM_001159487
GOTERM_BP_FAT	GO:0001568~blood vessel development	8	0,029465558	NM_010828, NM_009129, NM_010728, NM_009370, NM_016907, NM_008021, NM_008002, NM_008700
GOTERM_BP_FAT	GO:0045944~positive regulation of transcription from RNA polymerase II	10	0,03073695	NM_010828, NM_008259, NM_153553, NM_015743, NM_153786, NM_007922, NM_009719, NM_008021, NM_008700, NM_010234

	promoter			
GOTERM_BP_FAT	GO:0001944~vasculature development	8	0,032991888	NM_010828, NM_009129, NM_010728, NM_009370, NM_016907, NM_008021, NM_008002, NM_008700
GOTERM_BP_FAT	GO:0044093~positive regulation of molecular function	9	0,033307463	NM_013598, NM_008970, NM_027106, NM_001122733, NM_013732, NM_007429, NM_009625, NM_028679, NM_008700
GOTERM_BP_FAT	GO:0051241~negative regulation of multicellular organismal process	5	0,033480694	NM_013732, NM_007429, NM_001159487, NM_028679, NM_008940
GOTERM_BP_FAT	GO:0030198~extracellular matrix organization	5	0,034542982	NM_212452, NM_010728, NM_009370, NM_016907, NM_007424
GOTERM_BP_FAT	GO:0060648~mammary gland bud morphogenesis	2	0,035991247	NM_008970, NM_008002
GOTERM_BP_FAT	GO:0042981~regulation of apoptosis	13	0,037220035	NM_007540, NM_001111030, NM_013598, NM_009129, NM_009370, NM_007429, NM_008700, NM_010828, NM_008102, NM_021451, NM_007702, NM_028679, NM_138313
GOTERM_BP_FAT	GO:0010604~positive regulation of macromolecule metabolic process	14	0,044706227	NM_008259, NM_013598, NM_153553, NM_001122733, NM_015743, NM_007922, NM_009719, NM_011307, NM_008700, NM_010234, NM_010828, NM_153786, NM_010663, NM_008021
GOTERM_BP_FAT	GO:0040012~regulation of locomotion	5	0,044980781	NM_007903, NM_009215, NM_009129, NM_199465, NM_008002
GOTERM_BP_FAT	GO:0019953~sexual reproduction	10	0,045995382	NM_025727, NM_013598, NM_001039385, NM_021879, NM_009370, NM_001122733, NM_009061, NM_001159487, NM_013498, NM_198418
GOTERM_BP_FAT	GO:0003013~circulatory system process	5	0,046238115	NM_007903, NM_013732, NM_007429, NM_008102, NM_010933
GOTERM_BP_FAT	GO:0008015~blood circulation	5	0,046238115	NM_007903, NM_013732, NM_007429, NM_008102, NM_010933
GOTERM_BP_FAT	GO:0048562~embryonic organ morphogenesis	6	0,04655517	NM_015743, NM_009370, NM_009323, NM_026571, NM_008002, NM_001159487
GOTERM_BP_FAT	GO:0045935~positive regulation of nucleobase, nucleoside, nucleotide and	12	0,046684792	NM_010828, NM_008259, NM_008970, NM_153553, NM_015743, NM_153786, NM_007922, NM_009719, NM_008021, NM_011307,

	nucleic acid metabolic process			NM_008700, NM_010234
GOTERM_BP_FAT	GO:0007276~gamete generation	9	0,049003144	NM_025727, NM_013598, NM_021879, NM_009370, NM_001122733, NM_009061, NM_001159487, NM_013498, NM_198418
